# Single Dose Novel *Salmonella* Vaccine Enhances Resistance against Visceralizing *L. major* and *L. donovani* Infection in Susceptible BALB/c Mice

**DOI:** 10.1371/journal.pntd.0001406

**Published:** 2011-12-27

**Authors:** Juliane Schroeder, Najmeeyah Brown, Paul Kaye, Toni Aebischer

**Affiliations:** 1 Institute of Immunology and Infection Research, University of Edinburgh, Edinburgh, United Kingdom; 2 Centre for Immunology and Infection, Hull York Medical School and Department of Biology, University of York, York, United Kingdom; 3 Robert Koch-Institute, Berlin, Germany; Yale School of Public Health, United States of America

## Abstract

Visceral leishmaniasis is a major neglected tropical disease, with an estimated 500,000 new cases and more than 50,000 deaths attributable to this disease every year. Drug therapy is available but costly and resistance against several drug classes has evolved. Despite all efforts, no commercial, let alone affordable, vaccine is available to date. Thus, the development of cost effective, needle-independent vaccines is a high priority. Here, we have continued efforts to develop live vaccine carriers based on recombinant *Salmonella*. We used an *in silico* approach to select novel *Leishmania* parasite antigens from proteomic data sets, with selection criteria based on protein abundance, conservation across *Leishmania* species and low homology to host species. Five chosen antigens were differentially expressed on the surface or in the cytosol of *Salmonella typhimurium* SL3261. A two-step procedure was developed to select optimal *Salmonella* vaccine strains for each antigen, based on bacterial fitness and antigen expression levels. We show that vaccine strains of *Salmonella* expressing the novel *Leishmania* antigens LinJ08.1190 and LinJ23.0410 significantly reduced visceralisation of *L. major* and enhanced systemic resistance against *L. donovani* in susceptible BALB/c mice. The results show that *Salmonella* are valid vaccine carriers for inducing resistance against visceral leishmaniasis but that their use may not be suitable for all antigens.

## Author Summary

The leishmaniases are tropical diseases that affect the poorest of the poor. They are caused by *Leishmania* species, protozoan parasites transmitted by blood sucking insects and the visceral form of the disease is fatal. Vaccines that would tremendously boost disease control strategies need to be designed cost-efficiently and for the existing infrastructure. *Salmonella*-based live vaccines could fulfil these requirements as they can be cheaply produced on an industrial scale and the lyophilized product can be stored at room temperature and upon rehydration is ready for oral, needle-free application. *Salmonella*, like *Leishmania*, are intracellular pathogens that primarily target host macrophages. The bacteria induce a viscerotropic immune response. Herein lies a potentially significant advantage of using attenuated *Salmonella* as delivery vehicles for parasite antigens for vaccination against visceral leishmaniasis. We used *in vivo* inducible promoters and optimized expression systems to construct attenuated *Salmonella* carriers that deliver novel vaccine antigens and show a host protective effect in small rodent models of visceral leishmaniasis. These proof-of-concept studies should serve to further promote exploration of live *Salmonella* as a cost effective and widely applicable carrier for vaccination against leishmaniases.

## Introduction

The leishmaniases are regarded as neglected tropical diseases. The causative protozoan parasites are transmitted through the bite of sandfly vectors. Currently an estimated 12 million people are infected, while 350 million people in 88 countries worldwide are at risk to develop one of the diseases associated with *Leishmania* parasites (http://www.who.int/leishmaniasis/burden/en/; [1]). The most severe form is visceral leishmaniasis (VL; also known as kala azar in India) a disease that is fatal if untreated. An estimated 500 000 new cases and 50 000 deaths are reported every year, with 90% occurring in Bangladesh, Nepal, India, Sudan, Ethiopia and Brazil ([2]). VL caused by *L. infantum*/*chagasi* is zoonotic with dogs being the main reservoir; however, in areas endemic for *L. donovani* (e.g. India and Sudan) the disease is anthroponotic.

In many cases infection remains asymptomatic, most likely indicating immune control. However, patients with symptomatic VL experience fever, fatigue, weight loss and weakness often accompanied by hepato-splenomegaly and anaemia and, if untreated, may die from bacterial co-infections, internal bleeding and anaemia (reviewed by (2]). Chemotherapy is available, but due to high toxicity, adverse side effects and emerging parasite resistance, treatment options are limited [3]–[6]. Long treatment regimens and associated costs are additional critical factors preventing patient access and compliance. For example paromomycin, a newly registered drug, is given by intra-muscular injections over a period of 21 days. Though the cheapest drug available, treatment still costs between 5 and 10 US$ per course, making this drug too expensive in relation to household income [5]. This economic burden of treatment is likely to remain for the foreseeable future. Thus, developing a vaccine for VL (and indeed for other forms of leishmaniasis) is high on the agenda of the World Health Assembly (resolution EB118.R3, Geneva 05/07).

Vaccination is considered possible because of the efficacy of the century-old practice of leishmanization against old world cutaneous leishmaniasis (CL), a treatment that affords life long protection as proven during its large scale use to protect military personnel in Israel, Iran and the former Soviet Union [7]–[9]. However, in some individuals, development of non-healing lesions, exacerbation of chronic disease and immunosuppression as a result of this procedure has been observed [10]. The unsatisfactory safety profile, its questionable efficacy against infection with heterologous species and logistic hurdles render leishmanization problematic. Vaccines that relied on autoclaved or merthiolate-killed whole promastigotes formulated with or without Bacillus Calmette-Guerin as adjuvants were developed to remedy some of the shortcomings of leishmanization but a recent meta-analysis of clinical studies evaluating these vaccines did not support their efficacy [11].

Clinical testing of vaccines based on recombinant *Leishmania* antigens or fractionated parasite material is much less advanced, although numerous antigenic proteins have been shown to have vaccine potential in pre-clinical models (see reviews by [12]–[14]). These antigens were usually discovered by classical approaches, i.e. by screening with immune or hyperimmune sera from patients or infected animals. Antibody reactivity may not be an ideal criterion since protection is cell mediated and is thought to depend on both CD4^+^ and CD8^+^ T lymphocytes [15]–[18]. More recently, however, parasite genome information has become available and vaccine-antigen discovery exploiting this information has been promoted [19]. Recombinant DNA technology enables the formulation of subunit vaccines consisting of one or few specified antigens as DNA- and vectored vaccines, the latter exploiting viruses or bacteria as vaccine vehicles (summarised by [12], [20], [21]). Indeed, expressed sequence-tag based vaccine antigen discovery has been explored [22]. However, of 100 ORFs tested only 14 showed detectable protective effects when tested in a high dose infection model of murine CL. This was probably not surprising given that gene expression is regulated mainly post transcriptionally in *Leishmania* and suggests a need to improve sequence selection criteria.

Here, we adapted a reverse vaccinology [23] approach to define novel candidate vaccines, starting from proteomic data sets that were generated recently [24] and ignoring whether or not proteins would be recognized by sera from infected hosts. Moreover, we optimized recombinant attenuated *Salmonella* as a vaccine carrier platform since they had been explored before as vectors for anti-*Leishmania* vaccines [25]–[27] and have already been developed for vaccination purposes in humans [28]–[30].

## Materials and Methods

### Mice

Female BALB/c mice were purchased from Harlan UK, Charles River UK or bred and maintained under specific pathogen-free conditions in individually ventilated cages in the animal facilities of the School of Biological Sciences at the University of Edinburgh and the University of York. Animals were used at 6–9 weeks of age and were age matched within each experiment. All animal experiments adhered to the UK Animals (Scientific Procedures) Act 1986 and were conducted under Project Licenses granted by the UK Home Office and with local ethical approval (License # PPL 60/03581 to TA and PPL 60/03708 to PK).

### Construction of expression plasmids

To inducibly express antigens on the surface of *Salmonella*, the *E. coli* adhesin involved in diffuse adherence (AIDA) autotransporter system was adapted and a variant of plasmid pKRI143 [31] was constructed, pAIDA0, as previously described [32]. Briefly, the sequence encoding cholera toxin B subunit signal peptide was followed by *Spe*I/*Bgl*II sites for in frame directional cloning of ORF of interest fused with downstream sequences coding for a hemagglutinin epitope (HA)-tag and the transporter domain of AIDA, all under the control of the *in vivo* inducible Mg^2+^ responsive P_pagC_ promoter [33].

Vaccine antigen ORFs encoding *L. donovani* KMP-11 (LinJ35_V3.2260), ORF LinJ08.1190 (LinJ08_V3.1190), ORF LinJ09.1180 (LinJ09_V3.1180), ORF LinJ23.0410 (LinJ23_V3.0420), ORF LinJ25.1680 (LinJ25_V3.1670) and ORF LinJ35.0240 (LinJ35_V3.0140) were amplified from *L. donovani* (MHOM/INI/03BHU-55) genomic DNA using primers shown in table 1. ORF nomenclature and accession numbers are indicated in Table 2. Amplifications were carried out with the Platinum® *Pfx* DNA Polymerase kit (Invitrogen). PCR products were digested with *Spe*I and *Bgl*II and cloned into the equally digested pAIDA0 for transformation into SL3261 and *E. coli* JK321(UT5600 *zih*::Tn*10 dsbA*::*kan*) [34], respectively.

**Table 1:** List of primers used for amplifying antigens from parasite DNA.

Antigen	Expression system	Sequence 5′→3′(enzyme restriction sites are shown in bold)	
KMP-11	SurfacepsVAC	F	GATCAA**ACTAGT**GCCACCACGTACGAGGAG
		R	GATCAA**AGATCT**CTTGGATGGGTACTGCGCAGCC
	CytosolpcVAC	F	GATCAA**CATATG**GCCACCACGTACGAGGAG
		R	GATCAA**GGATCC**GTCGATTACTTGGATGGGTACTGCGCAGCC
LinJ08.1190	SurfacepsVAC	F	GATCAA**ACTAGT**TCTCAGCAGCTCGCCTTCC
		R	GATCAA**AGATCT**CGGGTGGCTGTCGTCGGCGGC
	CytosolpcVAC	F	GATCAA**CATATG**TCTCAGCAGCTCGCCTTCC
		R	GATCAA**GGATCC**GTCGATTACGGGTGGCTGTCGTCG
LinJ09.1180	SurfacepsVAC	F	GATCAA**ACTAGT**TCCCCTCTGCAGCAGGCACGCTGG
		R	GATCAA**AGATCT**CACTTTTCGGGAAAAACCAGTG
	CytosolpcVAC	F	GATCAA**CATATG**TCCCCTCTGCAGCAGGCACGCTGG
		R	GATCAA**GGATCC**GTCGACTACACTTTTCGGGAAAAACC
LinJ23.0410	SurfacepsVAC	F	GATCAA**ACTAGT**CTTCACTTCCCCATTTCGCCC
		R	GATCAA**AGATCT**CCAGCCGCGGTGATAGAGG
	CytosolpcVAC	F	GATCAA**CATATG**CTTCACTTCCCCATTTCGCCC
		R	GATCAA**GGATCC**GTCGATTACCAGCCGCGGTGATAGAGG
LinJ25.1680	SurfacepsVAC	F	GATCAA**ACTAGT**TCGTCCGAGGTTGCGATTCAGC
		R	GATCAA**AGATCT**CTGCTGCTGCTTCTCCGG
	CytosolpcVAC	F	GATCAA**CATATG**TCGTCCGAGGTTGCGATTCAGC
		R	GATCAA**GGATCC**GTCGACTACTGCTGCTGCTTCTCC
LinJ35.0240	SurfacepsVAC	F	GATCAA**ACTAGT**CTGCGCCACTCGCTGCTTCG
		R	GATCAA**AGATCT**CCACCAGGCTGCCTTGCGGATGC
	CytosolpcVAC	F	GATCAA**CATATG**CTGCGCCACTCGCTGCTTCG
		R	GATCAA**GGATCC**GTCGACTACCACCAGGCTGCCTTGC

**Table 2:** Nomenclature and accession numbers of genes encoding the novel antigens investigated.

In manuscript	In TriTrypDB1)(version 22.06.2011)	NCBI Reference sequenceAcc. Number
*LinJ.08.1190*	*LinJ.08.1190*	XM_001463411.1
*LinJ.09.1180*	*LinJ.09.1180*	XM_001463538.1
*LinJ.23.0410*	*LinJ.23.0420*	XM_001465681.1
*LinJ.25.1680*	*LinJ.25.1680*	XM_001466162.1
*LinJ.35.0240*	*LinJ.35.0140*	XM_001468797.1

1) Aslett et al. **TriTrypDB: a functional genomic resource for the Trypanosomatidae** Nucleic Acids Research 2010 38(Database issue):D457–D462; doi:10.1093/nar/gkp851.

To differentially regulate protein expression levels, point mutations were introduced into the Shine-Dalgarno ribosomal binding sequence (RBS; underlined) using site directed mutagenesis. Forward primer for RBS3 (5′-GATCAA**TCTAGA**TTTAAGAAGCAGATATACATATGATTAAATTAAAATTTGGTG-3′), RBS4 (5′-GATCAA**TCTAGA**TTTAAGAAGGGAATATACATATGATTAAATTAAAATTTGGTG-3′) and RBS5 (5′-GATCAA**TCTAGA**TTTAAGAAAGAAATATACATATGATTAAATTAAAATTTGGTG-3′) were designed to amplify the cholera toxin signal peptide, HA-tag and antigen while simultaneously introducing the mutated Shine-Dalgarno sequence upstream of the signal peptide. The resulting PCR product was *Spe*I/*Bgl*II digested and re-ligated into pAIDA-Antigen. All resulting surface expression plasmids were subsequently named psVAC[# of RBS mutation]-antigen.

For expression of antigens in the salmonella cytosol *L. donovani* ORFs were amplified using primers described in Table 1. Resulting PCR products flanked by 5′ *Nde*I and 3′ *BamH*I sites were digested and first cloned downstream of a P_pagC_ promoter into a pBR322-derived plasmid series already containing mutated Shine-Dalgarno sequences (RBS1 – AGGAA, RBS2 – GGGAA and RBS3 – AGCAG) described in [35] for transformation into SL3261. The resulting plasmids were subsequently named pcVAC[# of RBS mutation]-antigen. Preparation of live vaccine stocks, immunizations and determination of bacterial fitness by *in vivo* colonisation have been performed exactly as described before [32].

### Purification of recombinant proteins

For generating recombinant proteins, *Leishmania* antigen ORFs were cloned into pET28a(+) (Novagen). All antigens were amplified using the *Nde*I and *BamH*I site containing primers described above. Recombinant proteins were purified as described previously [32]. KMP-11, the only soluble protein was directly purified on a Nickel column (1 ml, HisTrap FF, GE Healthcare). All other antigens formed inclusion bodies which needed to be isolated and dissolved prior purification under denaturing conditions with an on-column refolding step [32].

Recombinant protein containing fractions eluted from columns (see Fig. S1) were pooled and protein concentrations determined using amidoblack [36]). Proteins LinJ08.1190, LinJ09.1180, LinJ23.0410 and LinJ25.1680 became insoluble when imidazole was removed; hence 50 µl/well of a 50 µg/ml protein eluate was used to coat 96-well plates (MaxiSorb, Nunc) for ELISA. Plates were sealed and stored at 4°C until needed. For T cell re-stimulation assays imidazole was removed by dialysis against TBS/150 mM NaCl and subsequently concentrated by ultrafiltration using Centricons® (Millipore) of appropriate pore size.

### Enzyme-linked immunosorbant assay

ELISA for antigen-specific antibodies of different isotypes (IgG1 and IgG2a) from mouse serum has been performed as previously described [32]. In brief, serial dilutions of individual sera were analysed. To estimate relative antibody concentrations, titers were determined corresponding to the value of the serum dilution giving a half maximal ELISA signal.

### *L. major* infection and determination of parasite burden in limiting dilution assays

*L. major* promastigotes were grown in semi-defined medium until late stationary phase was reached. Two million parasites were injected into the left hind footpad and lesion size was measured as the difference in thickness between infected and uninfected footpad using a calliper. For determination of parasite numbers in organs mice were sacrificed by cerebral dislocation and organs (spleen, draining lymph node, footpad) were removed and homogenized. The single cell suspensions were adjusted to equal volumes and subjected to serial dilutions in 96-well tissue culture plates filled with SDM medium [24] supplemented with 20 µg/ml hygromycin and 50 µg/ml kanamycin, which was carried out in quadruplets. After 14 days at 27°C, parasite growth was scored microscopically and parasite load in the infected organs was calculated using the dilution where at least 2 of 4 wells (>37.5%) were positive [37]. This dilution was multiplied by the total volume (in multiples of 0.1 ml) to derive the total number of parasites per organ.

### Determination of hepato-splenomegaly and *L. donovani* burden in impression smears

Mice were killed by cervical dislocation and livers and spleens were removed and weighed. The body-mass index (BMI) was calculated as the organ weight in percentage of body weight. To determine parasitic burden in spleen and liver, impression smears were prepared on microscopic glass slides, fixed in methanol and stained with Giemsa. The number of parasites per 1000 host cell nuclei was counted using a light-field microscope and an immersion oil lens. Leishman-Donovan units (LDU) were calculated by multiplication of the number of parasites/1000 nuclei with the organ weight [38].

### Histopathological analysis of hepatic response to *L. donovani*

Liver sections were processed for immunohistochemistry as described in detail elsewhere [39]. Briefly, confocal microscopy was performed on acetone fixed 8 µm frozen sections stained with Alexa 488-conjugated F4/80 (eBioscience, United Kingdom and purified rabbit anti-mouse inducible nitric oxide synthase (iNOS) (Abcam, United Kingdom) detected with donkey anti-rabbit Alexa 647. Sections were counterstained with 4′,6′-diamidino-2-phenylindole (DAPI), and mounted in Pro-Long Gold antifade (Invitrogen) for examination on a LSM META 510 confocal microscope (Zeiss). Quantification of NOS2 staining was performed on randomly selected fields for each mouse, using Adobe Photoshop CS3 to determine the area of iNOS reactivity (as number of positively stained pixels) relative to total granuloma area (as pixels stained with F4/80). Granuloma maturation was assessed from hematoxylin-eosin (H&E)-stained tissue sections as described elsewhere [39].

### Statistical analysis

Statistical analysis was performed using GraphPad Prism Program (Version 4.0, GraphPad Software, San Diego, California). Depending on data passing normality tests, ANOVA was performed with appropriate post-tests for pairwise comparisons or Mann-Whitney tests were computed. P values less than 0.05 were considered significant.

## Results

### *In silico* selection of novel antigen candidates from *Leishmania*

**Figure 1:**
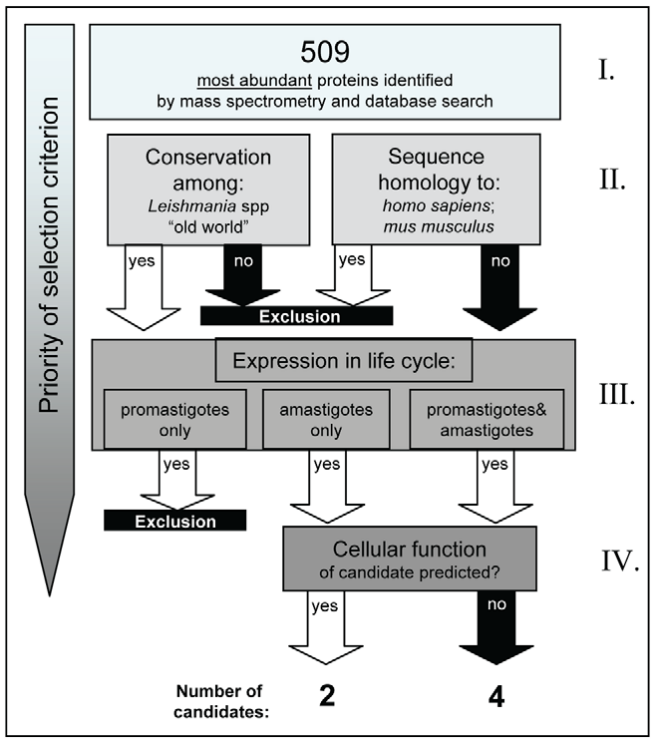
Schematic overview of criteria applied in antigen selection process. I: *Abundance*: A proteomic data set (c.f. Table S1 in ref. [24] consisting of 509 different proteins representing the most abundant proteins of *L. mexicana* was used as a starting set describing potential vaccine antigens. II: *No sequence homology to target vaccination populations* and *conservation within* Leishmania *spp.:* these criteria can be applied more or less stringently, we applied stringent thresholds for selecting against homologous proteins by focusing only on hypothetical conserved proteins (c.f. Table S1 in ref. [24] that at time of selection included LinJ09.1180) whereby the number of candidates was reduced to 185 at this step of selection. III: *Life-cycle stage occurrence*: proteomic evidence for expression of candidates in amastigotes or throughout the life cycle was required reducing the candidate list to 39 proteins. Preference was given in step VI to a set of six based on likelihood to be expressible as AIDA autotransporter fusion proteins in salmonella carriers and by re-iterating the selection based on conservation within the genus resulting in candidates with sequence identity >78%.

For the selection of novel antigen candidates, we conducted a bioinformatic analysis of a proteomic dataset that compared the proteomes of pro- and amastigote stages of *L. mexicana* [24]. This data set was chosen because to date this is the only dataset containing information on truly intracellular parasites and because a comparison with data from a proteomic analysis of *L. donovani* axenic amastigotes suggested a very high degree of overlap with respect to abundant proteins [24]. From a total of 509 proteins that reflect the set of highly abundant proteins, we selected five novel antigen candidates based on abundance, conservation throughout the genus and lack of homologies to host proteins (Figure 1). These criteria and, in addition, predicted subcellular localization were found before to be valuable to identify antigens for induction of protective T cell responses from complex organisms, operationally defined here as expressing ≫10^3^ different protein antigens, e.g. to select antigens for vaccines against *Helicobacter pylori* [40]. A further, *Leishmania*-relevant criterion was the expression of the potential antigen in appropriate life cycle stages. Preference was given to proteins expressed in the disease-causing intracellular amastigote stage, but, since early stages of infection after transmission of promastigotes were also considered relevant, antigen expression in both life cycle stages was not an exclusion criterion. Four selected antigens were present in the proteomic datasets of both stages while the LinJ23.0410 corresponding protein was present only in the amastigote dataset. Homologues of the encoding genes were found in all cases in *L. major*, *L. infantum*, *L. donovani* and *L. braziliensis* genomes with a very high degree of conservation (ranging from 78.9% to 95.8% identity of amino acid sequence, increasing to 87.6% to 99% when including conserved substitutions). Sequence homologies to proteins of mouse and human (human as final target and mouse as a model host) were excluded by BLAST searches. This approach was biased and preferentially excluded similar sequence-dependent epitopes. It was used here because it was assumed to enhance the likelihood of antigens to be recognized by T cells as “foreign” and to reduce the risk of potential autoimmune sequelae.

Novelty and expressability in our salmonella expression systems were additional final selection criterion but we also included the well characterized antigen KMP-11 as a reference vaccine antigen. This antigen has been shown to be protective against *L. donovani*, when administered as a DNA vaccine [41], [42].

### Generation and optimization of *S. typhimurium* vaccine carrier strains

**Figure 2:**
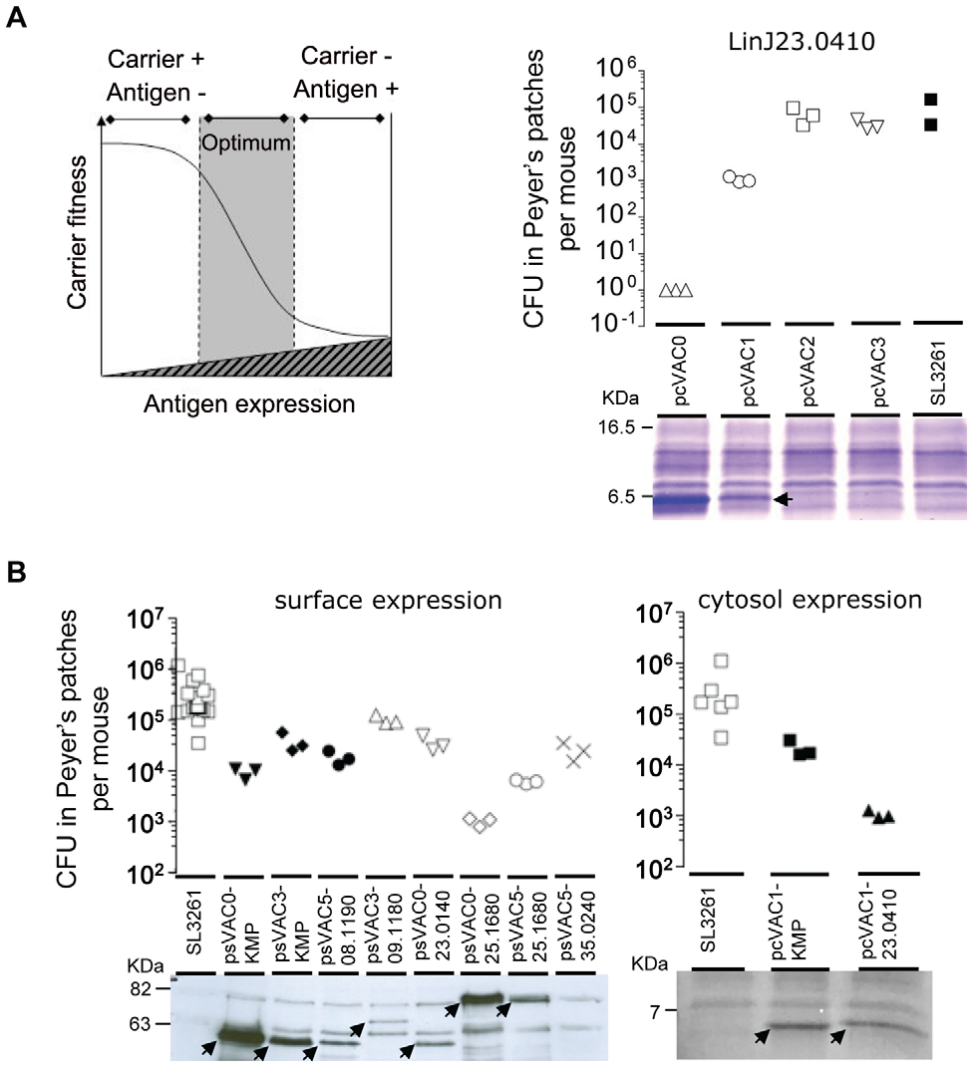
Two step strategy for the selection of vaccine strains for *in vivo* testing. Candidate antigen expression and ability to colonize Peyer's patches after intragastric inoculation was determined experimentally to select optimal strains. Expression of the HA-tagged antigens was assessed by SDS-PAGE in lysates of vaccine strains grown under low Mg^2+^ conditions to mimic the in vivo intraphagosomal environment that activates the P_pagC_ promoter. Vaccine antigen expression was visualized by coomassie stain (cytosol expressing strains) or by Western blot analysis using an anti-HA tag antibody (surface expressing strains). (A, left panel) A schematic plot is shown to illustrate how the degree of foreign antigen expression reduces bacterial fitness and how both factors influence the immunogenicity of a carrier strain. (A, right panel) Real data example of fitness as a function of antigen expression using SL3261 expressing increasing amounts of LinJ23.0410 in the cytosol. (B) Characteristics of bacterial fitness and recombinant protein produced by final selected strains with surface (left panel) or cytosolic (right panel) expression.

Subcellular localization and protein amount are not only useful criteria to select T cell vaccine antigens, they are also crucial parameters to consider in the construction of recombinant live vaccine carriers - such as bacteria - to induce antigen-specific cell mediated immunity [43], [44]. Thus, two expression systems were adapted that directed antigens either to the cytosol or the surface of *Salmonella* and allow induced expression via the *in vivo* inducible promoter P_pagC_. We choose to control antigen production at the translational level and introduced a set of point mutations into a canonical ribosomal binding site (RBS) creating a set of four plasmid cassettes each for cytosolic and surface antigen expression. These mutations resulted in staggered protein expression levels when *Salmonella* strains carrying the respective plasmids were grown under conditions that activate the P_pagC_ promoter (Figure S2). Heterologous protein expression can greatly reduce fitness of the carrier bacteria *in vivo*, thereby critically affecting the amount of total antigen delivered to the immune system and thus vaccine immunogenicity. This relationship is schematically shown in Figure 2A (left panel) and, as an example, is shown for vaccine strains engineered for cytosolic expression of LinJ23.0410 (Figure 2A, right panel). Colonisation of the Peyer's patches seven days after oral administration of 10^9^ CFU was determined as a measure of bacterial fitness. Expression of LinJ23.0410 was clearly negatively correlated with the number of CFU found in Peyer's patches, i.e. vaccine strain fitness. Use of a canonical, non-mutated RBS (RBS0) resulted in high amounts of protein but greatly reduced bacterial fitness. Introduction of point mutations (RBS1, 2, 3) lowered expression levels from intermediate (RBS1) to very low (RBS2 and 3) which brought fitness back to the level of the empty carrier strain (Figure 2A right panel). A reduction of bacterial fitness far below 10^4^ CFU in this assay, based on past experience (JS and TA unpublished), rendered vaccine strains non-immunogenic with respect to the recombinantly expressed antigen. Thus, out of 48 bacterial strains constructed and evaluated as shown for the example above, 10 strains were selected for further testing. Their respective fitness and antigen expression characteristics were as shown in Figure 2B.

Interestingly, antigens LinJ08.1190, LinJ09.1180, LinJ25.1680 and LinJ35.0240 could not be expressed in the cytosol (data not shown) but vaccine strains could be obtained, with the exception of LinJ35.0240, when the antigens were targeted to the bacterial surface. In consequence, only two vaccine strains expressing the antigens KMP-11 and LinJ23.0410 cytosolically could be included in the panel (Figure 2B, right panel). In addition, eight surface expression strains were selected (Figure. 2B, left panel). Surface expression of antigen LinJ35.0240 could not be detected via western blot despite a clear influence on bacterial fitness (Figure 2B left panel). Based on the latter, it was therefore decided to include psVAC5-35.0240 as an example for the respective antigen.

### Protection of mice against *L. major*

All selected strains were next tested *in vivo* for their ability to protect BALB/c mice against visceralising *L. major* infection. These mice are highly susceptible to *L. major* infection, and have been suggested to provide a good mouse model for VL. Mice were vaccinated with a single dose of *Salmonella* vaccine strains, the carrier control SL3261 or treated with PBS. Mice were subsequently challenged with 2×10^6^ late-stationary phase *L. major* promastigotes into the left hind footpad. Lesion size was monitored over a course of several weeks after which mice were randomized and selected for analysis of parasitic burden in footpad, lymph node and spleen.

A pilot study involving all 10 selected vaccine strains showed that vaccination with *Salmonella* carrying antigens LinJ08.1190 and LinJ23.0410 reduced lesion size and parasitic burden compared to the controls (see Figure S3). Interestingly, vaccination with antigen LinJ25.1680 expressing *Salmonella* exacerbated disease while the other vaccines including the KMP-11-expressing strains had no effect on disease progression compared to controls (Figure S3).

**Figure 3:**
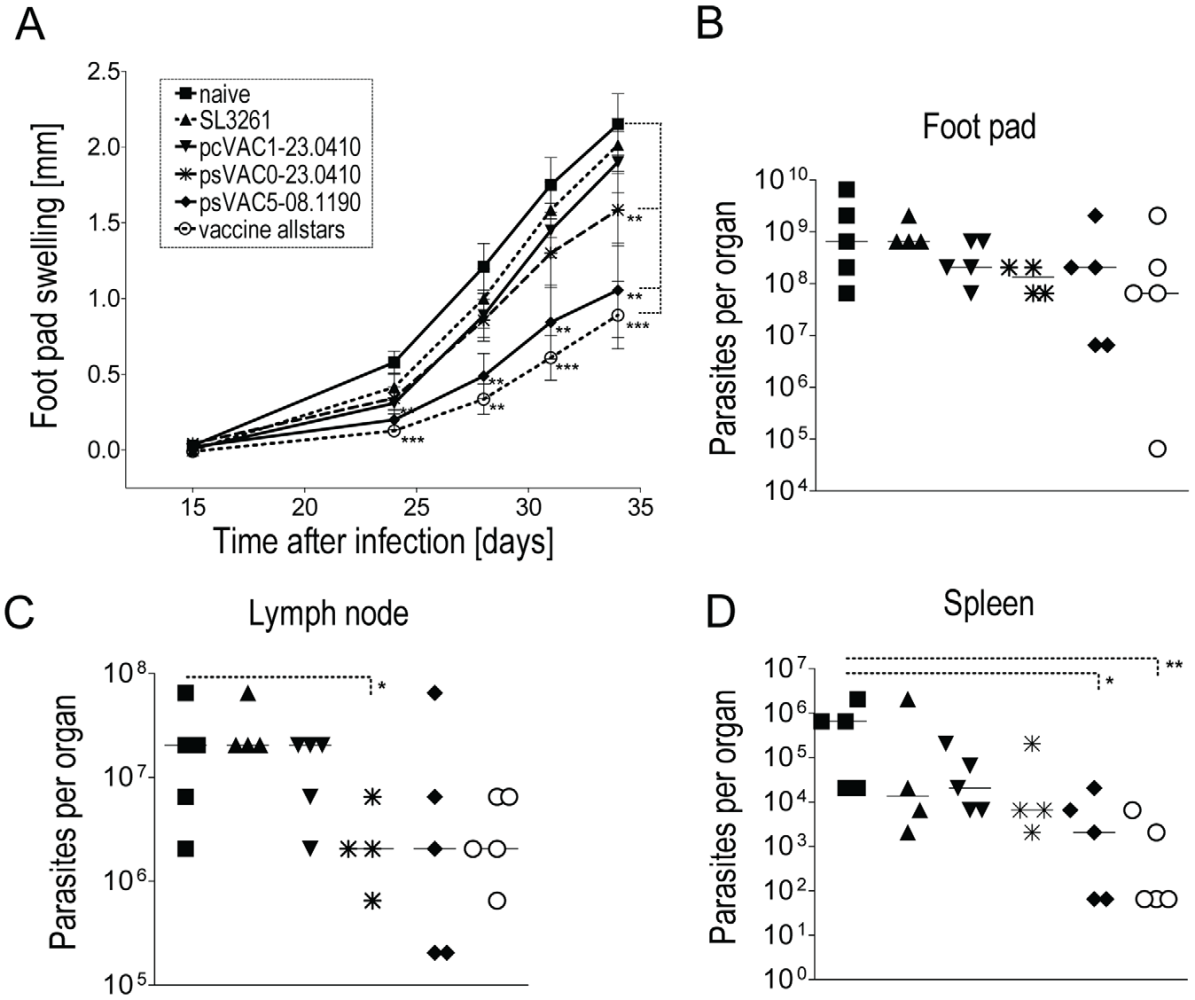
Vaccination significantly reduces *L. major* infection in BALB/c mice. Ten mice per group were immunised intra-gastrically with 10^10^ CFU of salmonella vaccine strains and infected 9 weeks later with 3×10^6^ stationary-phase *L. major* promastigotes into the left hind footpads. Footpad swelling (A) was monitored with a calliper using the uninfected right foot for reference. Values represent the mean ± standard error of the mean swelling per group and time point. Asterisks denote statistically significant differences (* P≤0.05, ** P≤0.01, *** P≤0.001; two tailed Mann-Whitney U test). Five weeks after infection 5 mice of each group were randomly selected and killed to determine parasite burden in footpads (B), draining lymph nodes (C) and spleens (D) by limiting dilution culture assay. The detection limit of the respective assays based on starting dilution was 65 parasites for spleen and 6500 for lymph node or footpad. The values of the burden per organ of all individual mice and median burden per group are shown. The asterisks denote statistically significant differences (* P≤0.05, ** P≤0.01; two-tailed Mann-Whitney U test). Error bars show standard error of the mean (SEM).

Thus, the presumably protective vaccine strains psVAC5-08.1190, pcVAC1-23.0410 and psVAC0-23.0410 as well as a mixture of these (from hereon named ‘vaccine allstars’), were further evaluated (Figure 3). Vaccination, especially with psVAC5-08.1190 and vaccine allstars, significantly delayed the onset and progression of footpad swelling in mice challenged nine weeks later (Figure 3A). Five weeks after infection, five animals per groups were selected randomly and parasitic burden in spleen, popliteal lymph node and footpad was determined. Parasite numbers in footpads and lymph nodes were not significantly different in the vaccine groups (Figure 3B, C) although a trend towards lower burdens was notable in mice vaccinated with psVAC0-23.0410, psVAC5-08.1190 and vaccine allstars (Figure 3B, C). The discrepancy between lesion size and parasite burden was surprising but is not without precedence. The inverse situation has been described in murine *L. major* infection when analysing TNR-p55 receptor deficient mice [45] or when mapping susceptibility loci [46], [47]. However, mechanisms are currently not fully understood. The parasitic burden in the spleen was assessed as a surrogate marker of protection against visceral leishmaniasis. Immunisation with the psVAC5-08.1190 and allstars vaccines significantly reduced parasite numbers in the spleen compared to challenged only mice and a similar trend was noted for the surface expressing psVAC0-23.0140 vaccine (Figure 3D). Of note, five animals amongst those vaccinated with psVAC5-08.1190 and vaccine allstars had no detectable parasites in the spleen (Figure 3D). Hence, a single oral dose of *Salmonella* vectored vaccines that delivered both LinJ08.1190 with LinJ23.0410 significantly reduced visceral *L. major* parasite burdens in these highly susceptible BALB/c mice.

### Protection of mice against *L. donovani*

Since conservation of the antigens among *Leishmania* species was a key selection criterion, we hypothesised that antigens which were protective against *L. major* would also protect against the causative agent of human VL, *L. donovani*. To test this hypothesis, we immunised BALB/c mice with strains psVAC5-08.1190 and vaccine allstars. *Leishmania* surface antigen KMP-11 had been shown to be protective against *L. donovani* in mice [42]. Therefore and despite its poor performance in previous experiments, *Salmonella* strain pcVAC1-KMP, expressing KMP-11 in the cytosol, was included together with the carrier strain SL3261 and sham-immunisation in this study. Mice vaccinated with a single oral dose were challenged intravenously with 3×10^7^ *L. donovani* amastigotes six weeks later. A characteristic for *L. donovani* infection in BALB/c mice is hepato-splenomegaly and the organ-specific control of the infection. Half of the mice were sacrificed on day 28 p.i., when liver parasite burden has usually reached its peak before the onset of self cure and when splenic parasite burden has begun to increase. The remaining animals were analysed at day 68 p.i. to assess long term control, particularly in the spleen.

**Figure 4:**
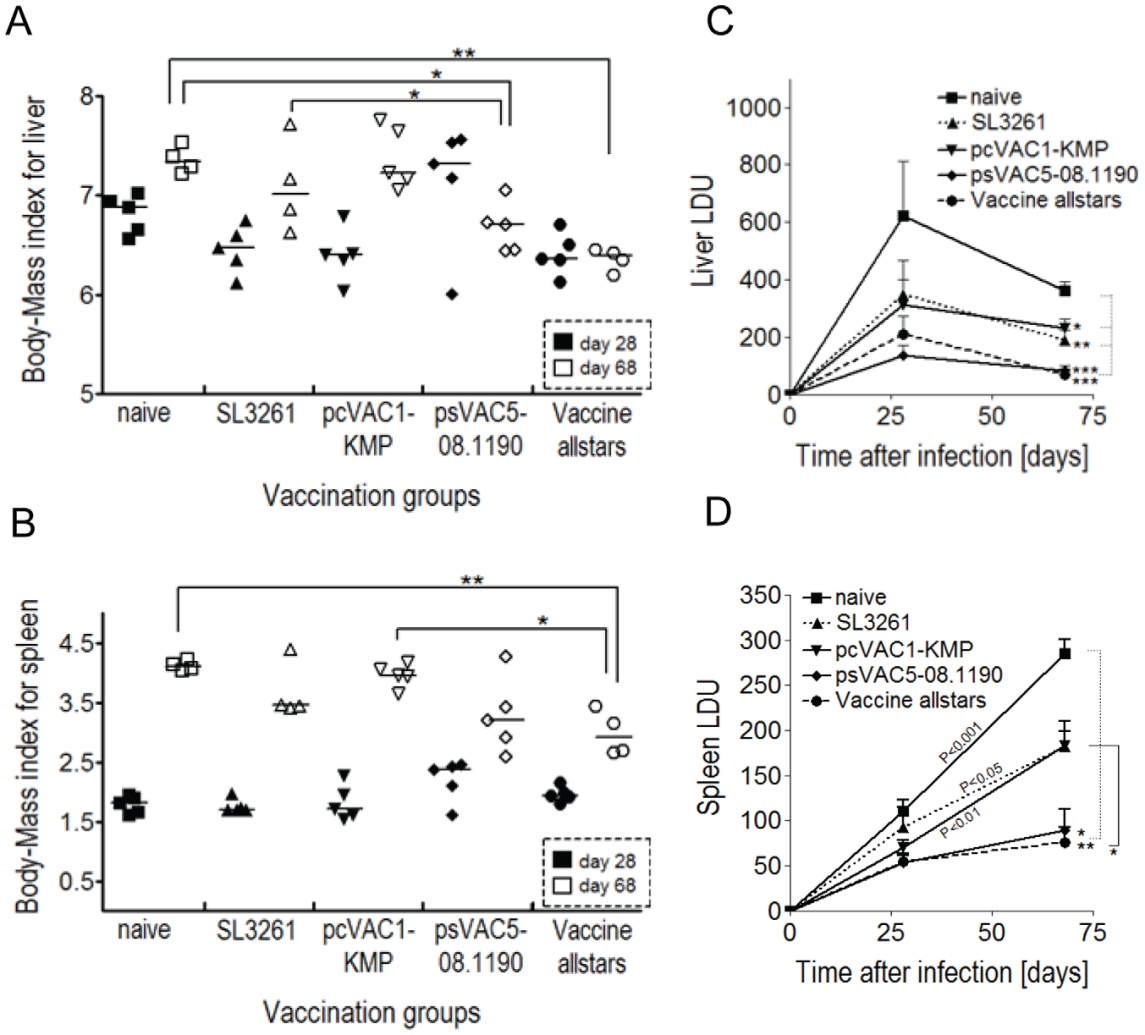
Vaccination significantly reduces *L. donovani* infection in BALB/c mice. Ten mice per group were immunised intra-gastrically with 10^10^ CFU salmonella vaccine strains and 6 weeks later infected with 3×10^6^ lesion-derived *L. donovani* amastigotes. Five mice per group were sacrificed at day 28 and five at 68 post infection. Animals were weighed and spleen and liver organ weights were determined to compute organ to body weight ratios for liver (A) and spleen (B). Giemsa stained impression smears of liver (C) and spleen (D) were assessed under the microscope and parasites per 1000 nuclei expressed as Leishman-Donovan Unit (LDU). * P≤0.05, ** P≤0.01, *** P<0.001; one-way ANOVA and Tukey post-test. Error bars show standard error of the mean (SEM).

An increased ratio of liver/spleen weight to body weight is an indirect measure of *L. donovani* infection induced inflammation and disease severity. Thus, body and organ weights were determined at necropsy (Table S1). The ratio for both liver (Figure 4A) and spleen (Figure 4B) increased between day 28 and day 68 in non-vaccinated animals and mice treated with either the carrier salmonella alone or the pcVAC1-KMP vaccine. In contrast, in animals vaccinated with psVAC5-08.1190 or the allstars vaccine, this ratio either increased less dramatically or not at all (Figure 4A, B). Mice immunized with psVAC5-08.1190 or the allstars vaccine had a mean liver parasite burden of 84.20±39.30 and 69.75±20.74 LDU, respectively at day 68 p.i. significantly reduced in comparison to the non-immunized group (361.0±66.79 LDU), the SL3261 carrier (189.0±63.79 LDU) or the pcVAC1-KMP treated animals (232.2±30.02 LDU; Figure 4C). Of note, the decrease noted after SL3261 treatment in comparison with the naïve controls was also significant (Figure 4C). The effects of the vaccines on splenic parasite burdens followed the same pattern (Figure 4D). Mice immunized with psVAC5-08.1190 or the allstars vaccine controlled parasite replication while numbers increased significantly between day 28 and 68 in all other study groups (Figure 4D). Immunisation with pcVAC1-KMP also did not protect mice from *L. donovani* infection and parasite burden increased over time (69.80±18.67 to 182.8±61.53), which was similar for SL3261 treated mice (Figure 4D).

In summary, a single oral dose of salmonella vectored vaccines delivering LinJ08.1190 and/or LinJ23.0410 significantly reduced hepato-splenomegaly and visceral infection in mice infected with *L. donovani*, the causative agent of human VL.

### Immune responses

**Figure 5:**
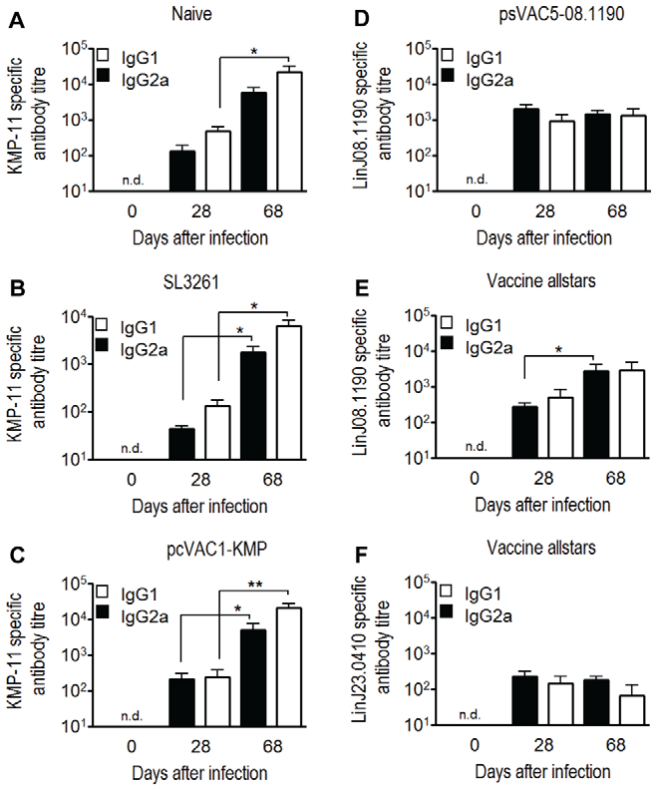
Analysis of antigen-specific humoral response in vaccinated *L. donovani* infected mice. IgG1 and IgG2a antibody isotypes specific for the respective vaccination antigen were determined in sera from each mouse individually by ELISA using plates coated with purified antigens KMP-11, LinJ08.1190 and LinJ23.0410. Titers of antibodies recognizing specific antigens of sera prepared at the indicted time points are shown from naïve (A), SL3261 carrier control (B) and pcVAC1-KMP vaccine strain (C) immunized animals or psVAC5-08.1190 vaccine (D) or vaccine allstars (E, F) treated mice. Values represent mean titers calculated from individually tested sera. The test had a sensitivity to detect antibodies with titers of <20. Error bars show standard error of the mean (SEM). n.d. not detected. Statistical significance: * P<0.05, ** P<0.01 by two-tailed Mann-Whitney U test.

To assess immune responses during vaccination and infection, we measured antigen-specific antibody isotype titres as a surrogate of the underlying CD4^+^ T cell response, given the known correlation between IL-4 and IgG1 responses and between IFNγ and IgG2a [48]. Serum was assessed in vaccinated mice four weeks after immunisation and on day 28 and 68 post infection with *L. donovani* to test for antigen-specific antibodies. Four weeks after vaccination but before infection, vaccine antigen-specific antibody titers were below the limit of detection (Figure 5A–F). In agreement with the fact that KMP-11-specific antibodies are produced during human VL [49], infected non-vaccinated mice or SL3261 carrier immunized mice generated anti-KMP-11 antibodies (Figure 5A, B). This anti-KMP-11 response was very similar in the pcVAC1-KMP vaccinated group (Figure 5C). In contrast, vaccines expressing LinJ08.1190 and/or LinJ23.0410 primed animals for the production of specific antibodies that became detectable after the boosting infection on day 28 and 68 post infection (Figure 5D–F) but no antibodies against the respective recombinant proteins were detectable by ELISA (detection limit of assay was at titers ≤20) during infection in naïve, SL3261 or pcVAC1-KMP treated animals (not shown). This indicated that LinJ08.1190 and/or LinJ23.0410 were not naturally immunogenic during infection of BALB/c mice.

**Figure 6:**
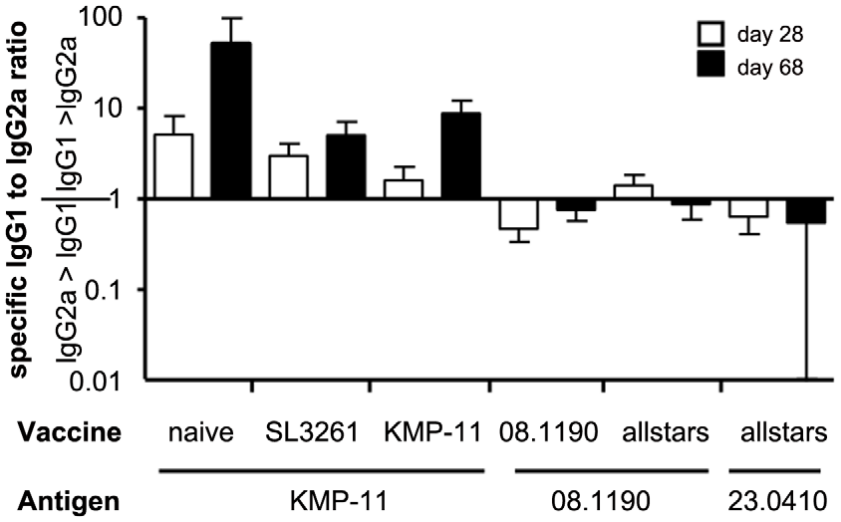
Changes in IgG1 to IgG2a ratios in the course of *L. donovani* infection. The ratios of antigen-specific IgG1 and IgG2a serum titres (as shown in Fig. 5) were calculated for sera of each individual mouse within their respective vaccination group for both time points (day 28 and day 68). Error bars show standard error of the mean (SEM).

Next, the ratios of vaccine antigen-specific IgG1 and IgG2a were calculated for each mouse and time point (Figure 6) to seek evidence for a bias in type 1 vs. type 2 immune response. Over the course of infection significant and different skewing was noted between the treatment groups. Anti-KMP-11 IgG1 to IgG2a ratios were above 1 in pcVAC1-KMP vaccinated mice which was therefore not different from the response to KMP in infected only or SL3261 vaccinated mice. In comparison, anti-vaccine antigen specific IgG1 to IgG2a ratios, however, were significantly different in sera from psVAC5-08.1190 or allstars vaccinated mice with values around 1 or below (Figure 6; p<0.05).

**Figure 7:**
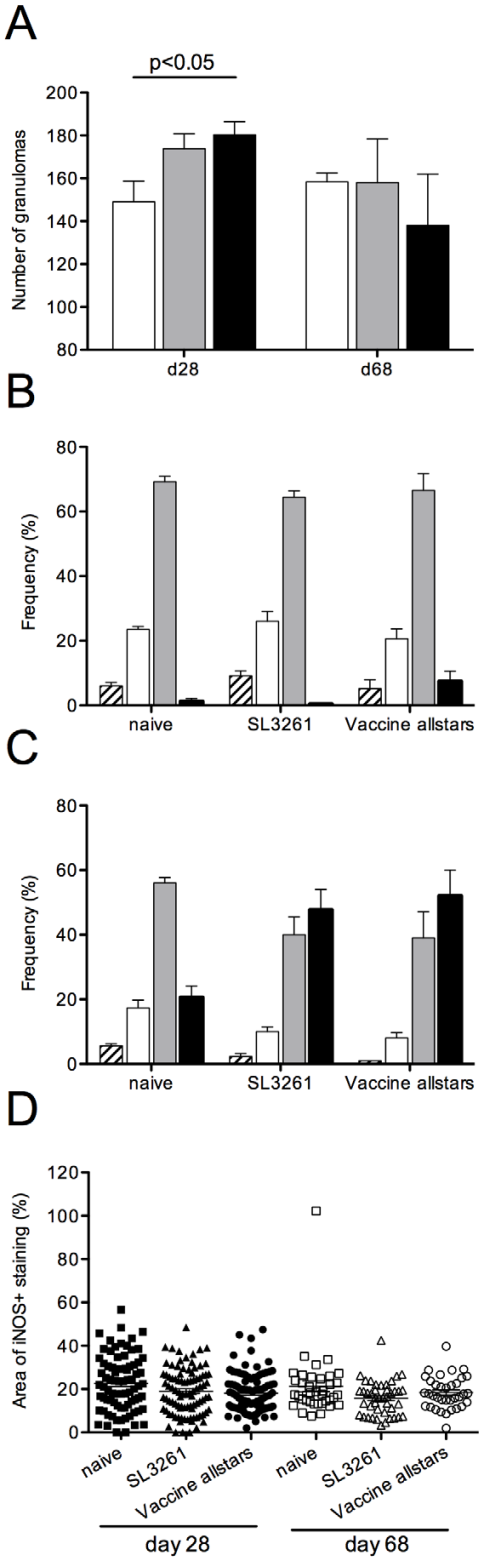
Hepatic granulomatous response in vaccinated mice. Liver sections were taken from mice used to assess parasite burden (Fig. 4) and stained for iNOS and F4/80. Nuclei were counterstained with DAPI. (A). Number of granulomas per 50 random fields of view (n = 5 mice per time point). (B and C) Granuloma maturation status was graded as described in Materials and Methods into no response (hatched bar), immature (open bar), mature (grey bar) and empty (black bar). Data were obtained from ∼50 granulomas per mouse and 4–5 mice at both day 28 (B) and day 68 (C) post infection. D. iNOS staining was assessed by determining the proportion of granuloma macrophage area that was stained for iNOS. Data are derived from between 35–90 granulomas from 5 mice per time point.

Finally, to assess the underlying cellular response in a more direct manner, we examined the level of granulomatous inflammation in infected mice that were either unvaccinated or had been vaccinated with control SL3261 *Salmonella* or with allstars (Figure 7). At day 28 p.i, there was a small but significant increase in the number of granulomas observed in the liver of allstars vaccinated mice (Figure 7A). We next measured the maturation stage of each granuloma, using established scoring criteria [39]. Granuloma maturation was similar between all groups of mice at day 28 p.i. (with a small but not significant trend towards enhanced maturation in allstars vaccinated mice). By day 68 p.i., however, mice vaccinated with either SL3261 or allstars showed enhanced granuloma maturation compared to non vaccinated mice. Although the results of this analysis are in keeping with the enhanced ability of these vaccinated mice to reduce parasite burden, it was not a sufficiently sensitive technique to discriminate between the resistance induced by SL3261 and allstars (c.f. Figure 4D). Finally, we measured the area within each granuloma that stained positive for iNOS, as one measure of functional capacity at these inflammatory foci. There were no significant differences in the iNOS response between vaccinated and non-vaccinated mice at either time point by this criterion (Figure 7D). Hence, the main tissue correlate of protection induced by allstars vaccination was an increase in the rapidity of granuloma formation, suggesting that vaccination may have heightened the frequency of CD4^+^ and/or CD8^+^ T cells able to facilitate this focal inflammatory response.

## Discussion

We had previously reported on the proteome of the intracellular amastigote stage of *L. mexicana* [24] which showed extensive overlap with proteins identified in *L. donovani* axenic amastigotes [50]. Because of this overlap, the former proteomic dataset was exploited here to adapt a reverse vaccinology approach to develop a vaccine against VL. We applied the criteria of protein abundance, within parasite genus conservation, and absence of homologous proteins in host organisms to select novel candidate vaccine antigens aimed to induce cellular immunity. These criteria may not be optimal though to select targets for inducing antibody-dependent immunity. Four of five selected candidates could be expressed in recombinant form and when delivered by recombinant *Salmonella* two reduced and one exacerbated disease progression in a murine *L. major* infection model. These results suggest that the frequency of identifying immunologically relevant proteins by this method is high and may well be superior to previous strategies that relied on mRNA expression and genome data for antigen selection [51], [52] with a hit frequency of ∼15%.

*Leishmania* like other kinetoplastids regulate gene expression mostly post-transcriptionally and mRNA abundance data alone may not be informative to predict protein abundance. However, as in shown in other systems [40] and *Leishmania* [53] actual protein abundance in amastigotes is highly relevant if the protein is to become a target of the immune response [54]. Analysis of the proteome data sets suggested that bias in codon usage indicates translational bias and therefore is highly correlated with protein abundance [24]. Hence, codon usage may be used to rank ORFs and serve as a substitute parameter for protein abundance in the absence of real protein expression data to refine pure in silico selection of candidate antigens. This becomes particularly relevant for selecting membrane proteins that are severely underrepresented in current proteomic data sets.

The two protective antigens, LinJ08.1190 and LinJ23.0410, expressed by *Salmonella* carriers were immunogenic in these vaccines yet, based on antibody responses, were not a target of the immune response to *L. donovani* infection, at least not in mice. This is noteworthy since many *Leishmania* vaccine antigens including KMP-11 currently favoured by other groups have been identified using sera from patients [55]–[58]. Our findings with the salmonella vectored KMP-11 vaccine suggest that these immunoselection approaches may introduce an extra hurdle for vaccine development since the natural antigen-specific response may be skewed and, possibly, even be disease exacerbating [59], [60]. The requirement for an additional type 1 immune response inducing adjuvants, IL-12, to achieve protective effects with a KMP-11 DNA vaccine in the murine *L. major* model [61] is in good agreement with this idea. Furthermore, a fusion protein called LEISH-F1 - also known as Leish-110F, Leish-111f or MML – was derived from the sequence of three immunoselected parasite antigens. LEISH-F1 is the most advanced protein-based subunit Leishmania vaccine in trial to date and has shown promising effects when tested in a therapeutic setting against human American CL [62]. However, this is not the case when used to prevent visceral canine disease after high dose experimental infection [63] or to treat naturally acquired VL in dogs [64]. In contrast, Leishmune®, a vaccine based on a glycoproteic fraction of *L. donovani* that was not immunoselected, is licensed for the prevention of canine VL in Brazil and has shown efficacy in the field [65]. Interestingly, the Leishmune® vaccine antigens are poorly recognized by sera from dogs suffering from VL and vaccination therefore is not interfering with sero-surveillance programs [65]. Thus, reverse vaccinology based approaches as presented here are likely to significantly broaden the choice of protective antigens.

A number of subunit vaccine delivery platforms, including purified proteins or mixtures of glycans and glycoproteins, recombinant DNA, viral and bacterial vectors have been evaluated experimentally in murine models of leishmaniases (review by [12]. However, very few have entered or passed clinical testing and amongst them no vectored vaccine. We have chosen *Salmonella* as a carrier since these bacteria had already been positively evaluated by several groups in experimental models of leishmaniases [25]–[27]. Moreover, they are being developed as recombinant carriers against a number of pathogens including *Helicobacter pylori*, Hepatitis B virus and *Plasmodium falciparum* [66], [67]. In the context of a major neglected disease such as VL, their main advantages are their excellent safety profile, simple and low-cost production at industrial scale, possibility to store as lyophilized product at room temperature, and oral application route, thus reducing the requirements for extensive infrastructure. In addition, *Salmonella* are potent inducers of long-lived cell-mediated immunity including CD8^+^ T cells [28], [68]. Induction of CD8^+^ T cells is particularly efficient by vaccines delivered by viral or bacterial carriers and may be a crucial characteristic of anti-Leishmania vaccines, since both CD4^+^ and CD8^+^ T cells are required for optimal anti-leishmanial immunity and granuloma formation [15]–[18]. While we do not yet have formal proof that our vaccines induced antigen-specific CD8^+^ T cells, bioinformatics analysis using CD8 T cell epitope/HLA-binding peptide prediction algorithms suggested epitopes presentable by major HLA alleles e.g. of human populations in VL endemic areas in India [69]. In the context of VL, *Salmonella* have the additional property to generate viscerotropic immune responses which may explain that the main protective effect was observed at the level of visceralizing infection in the *L. major* model. Moreover, depending on serovar, *S. enterica* exhibits broad or narrow host ranges. Serovar Typhimurium that was used here has the potential to deliver vaccine antigens in humans [70] as well as in dogs [71]–[73] while attenuated *S. enterica* Typhi can be engineered to deliver human vaccines [30], [66].

In summary, we report the identification of two novel candidate vaccine antigens against VL by reverse vaccinology and the optimized construction of live *Salmonella* carriers. These VL vaccines could potentially be used to combat VL in the zoonotic, as well as the anthroponotic cycle of the disease.

## Supporting Information

Figure S1 **Post-column fractions of purified his-tagged antigens.** His-tagged Leishmania antigens were expressed and purified from *E. coli* cell from inclusion bodies (LinJ23.0410 (A); LinJ08.1190 (B)) or lysates (KMP11 (C)) using nickel column liquid chromatography as described in Materials and Methods. Collected fractions were separated on SDS-Gels to determine yield and purity and selected fractions (box) were pooled and subsequently subjected to downstream processing such as dialysis and ultrafiltration.
(TIF)

Figure S2 **Two-step strategy for the selection of vaccine strains expressing cytosolic KMP-11 for** ***in vivo*** **testing.** Bacterial fitness (top panel) was determined as ability of the vaccine strain to colonize the Peyer's patches of mice seven days after single dose oral administration of 10^10^ CFU with KMP-11 expressing SL3261 (open symbols) or carrier SL3261 control (closed symbol; Schroeder and Aebischer (32)). For antigen expression (bottom panel), bacterial strains were grown under conditions mimicking the intraphagosomal environment (low Mg^2+^, Ca^2+^) and thus activating P_pagC_ promoter activated vaccine antigen expression. Translational efficiency depended on ribosomal binding site sequences that were the only difference between expression plasmids pcVAC0-3. 20 µg whole bacterial lysate per strain was loaded onto SDS gels and bands were visualized by coomassie stain. Black arrowhead indicates induced protein of interest (KMP-11).
(TIF)

Figure S3 **Pilot-study to identify vaccine strains protective against** ***L. major*** **infection.** Mice (10 per group) were immunised orally with a single dose of 10^10^ CFU of Salmonella vaccine strains carrying the indicated vaccine antigen expression plasmids or the carrier control SL3261. Twelve weeks later animals were challenged with 2×10^6^ *L. major* promastigotes into the left hind foot pad. Lesion size (A) was monitored with a calliper using the uninfected right foot as reference. Values represent mean swelling in mm and bars show standard errors of the mean (SEM). Asterisks denote statistical thresholds * P≤0.05, ** P≤0.01, *** P≤0.001 determined by two-tailed Mann-Whitney U test. Seven weeks after infection, three mice per group were randomly selected for determination of parasite burden in the foot pad (B), draining lymph node (D and spleen (C) were by limiting dilution assay as described in Materials and Methods. n.d. denotes not determined.
(TIF)

Table S1 **Organ and body weights were determined for each mouse in the respective groups immunized with SL3261 or SL3261 carrying the indicated vaccine antigen expression plasmid in comparison to a group of untreated mice.** Five mice per group per time point were killed by cervical dislocation and livers and spleens were removed and weighed. The body-mass indices (BMIs; see Fig. 4 in main manuscript) were calculated based on the mean weight values. n.d. = not done.
(XLS)

## References

[1] Herwaldt BL (1999). Leishmaniasis.. Lancet.

[2] Chappuis F, Sundar S, Hailu A, Ghalib H, Rijal S (2007). Visceral leishmaniasis: what are the needs for diagnosis, treatment and control?. Nat Rev Microbiol.

[3] Polonio T, Efferth T (2008). Leishmaniasis: drug resistance and natural products.. Int J Mol Med.

[4] Lira R, Sundar S, Makharia A, Kenney R, Gam A (1999). Evidence that the high incidence of treatment failures in Indian kala-azar is due to the emergence of antimony-resistant strains of *Leishmania donovani*.. J Infect Dis.

[5] Meheus F, Balasegaram M, Olliaro P, Sundar S, Rijal S (2010). Cost-Effectiveness analysis of combination therapies for visceral leishmaniasis in the Indian subcontinent.. PLoS Negl Trop Dis.

[6] Olliaro PL, Guerin PJ, Gerstl S, Haaskjold AA, Rottingen JA (2005). Treatment options for visceral leishmaniasis: a systematic review of clinical studies done in India, 1980–2004.. Lancet Infect Dis.

[7] Nadim A, Javadian E, Tahvildar-Bidruni G, Ghorbani M (1983). Effectiveness of leishmanization in the control of cutaneous leishmaniasis.. Bull Soc Pathol Exot Filiales.

[8] Khamesipour A, Rafati S, Davoudi N, Maboudi F, Modabber F (2006). Leishmaniasis vaccine candidates for development: a global overview.. Indian J Med Res.

[9] Khamesipour A, Dowlati Y, Asilian A, Hashemi-Fesharki R, Javadi A (2005). Leishmanization: use of an old method for evaluation of candidate vaccines against leishmaniasis.. Vaccine.

[10] Hosseini SM, Hatam GR, Ardehali S (2005). Characterization of Leishmania isolated from unhealed lesions caused by leishmanization.. East Mediterr Health J.

[11] Noazin S, Khamesipour A, Moulton LH, Tanner M, Nasseri K (2009). Efficacy of killed whole-parasite vaccines in the prevention of leishmaniasis: a meta-analysis.. Vaccine.

[12] Kedzierski L (2010). Leishmaniasis Vaccine: Where are We Today?. J Glob Infect Dis.

[13] Okwor I, Uzonna J (2009). Vaccines and vaccination strategies against human cutaneous leishmaniasis.. Hum Vaccin.

[14] Costa CHN, Peters NC, Maruyama SR, de Brito EC, de Miranda Santos IKF (2011). Vaccines for the Leishmaniases: Proposals for a Research Agenda.. PLoS Negl Trop Dis.

[15] Stager S, Alexander J, Kirby AC, Botto M, Rooijen NV (2003). Natural antibodies and complement are endogenous adjuvants for vaccine-induced CD8^+^ T-cell responses.. Nat Med.

[16] Basu R, Bhaumik S, Haldar AK, Naskar K, De T (2007). Hybrid cell vaccination resolves *Leishmania donovani* infection by eliciting a strong CD8^+^ cytotoxic T-lymphocyte response with concomitant suppression of interleukin-10 (IL-10) but not IL-4 or IL-13.. Infect Immun.

[17] Muller I, Kropf P, Etges RJ, Louis JA (1993). Gamma interferon response in secondary *Leishmania major* infection: role of CD8^+^ T cells.. Infect Immun.

[18] Gurunathan S, Sacks DL, Brown DR, Reiner SL, Charest H (1997). Vaccination with DNA encoding the immunodominant LACK parasite antigen confers protective immunity to mice infected with *Leishmania major*.. J Exp Med.

[19] Dumonteil E (2009). Vaccine development against *Trypanosoma cruzi* and Leishmania species in the post-genomic era.. Infect Genet Evol.

[20] Palatnik-de-Sousa CB (2008). Vaccines for leishmaniasis in the fore coming 25 years.. Vaccine.

[21] Coler RN, Reed SG (2005). Second-generation vaccines against leishmaniasis.. Trends Parasitol.

[22] Stober CB, Lange UG, Roberts MT, Gilmartin B, Francis R (2006). From genome to vaccines for leishmaniasis: screening 100 novel vaccine candidates against murine *Leishmania major* infection.. Vaccine.

[23] Rappuoli R (2000). Reverse vaccinology.. Curr Opin Microbiol.

[24] Paape D, Lippuner C, Schmid M, Ackermann R, Barrios-Llerena ME (2008). Transgenic, fluorescent *Leishmania mexicana* allow direct analysis of the proteome of intracellular amastigotes.. Mol Cell Proteomics.

[25] Yang DM, Fairweather N, Button LL, McMaster WR, Kahl LP (1990). Oral *Salmonella typhimurium* (AroA-) vaccine expressing a major leishmanial surface protein (gp63) preferentially induces T helper 1 cells and protective immunity against leishmaniasis.. J Immunol.

[26] McSorley SJ, Xu D, Liew FY (1997). Vaccine efficacy of Salmonella strains expressing glycoprotein 63 with different promoters.. Infect Immun.

[27] Lange UG, Mastroeni P, Blackwell JM, Stober CB (2004). DNA-*Salmonella enterica* serovar Typhimurium primer-booster vaccination biases towards T helper 1 responses and enhances protection against *Leishmania major* infection in mice.. Infect Immun.

[28] Salerno-Goncalves R, Wyant TL, Pasetti MF, Fernandez-Vina M, Tacket CO (2003). Concomitant induction of CD4^+^ and CD8^+^ T cell responses in volunteers immunized with *Salmonella enterica* serovar Typhi strain CVD 908-htrA.. J Immunol.

[29] Aebischer T, Bumann D, Epple HJ, Metzger W, Schneider T (2008). Correlation of T cell response and bacterial clearance in human volunteers challenged with *Helicobacter pylori* revealed by randomised controlled vaccination with Ty21a-based Salmonella vaccines.. Gut.

[30] Bumann D, Hueck C, Aebischer T, Meyer TF (2000). Recombinant live Salmonella spp. for human vaccination against heterologous pathogens.. FEMS Immunol Med Microbiol.

[31] Rizos K, Lattemann CT, Bumann D, Meyer TF, Aebischer T (2003). Autodisplay: efficacious surface exposure of antigenic UreA fragments from *Helicobacter pylori* in Salmonella vaccine strains.. Infect Immun.

[32] Schroeder J, Aebischer T (2009). Recombinant outer membrane vesicles to augment antigen-specific live vaccine responses.. Vaccine.

[33] Dunstan SJ, Simmons CP, Strugnell RA (1999). Use of in vivo-regulated promoters to deliver antigens from attenuated *Salmonella enterica* var. Typhimurium.. Infect Immun.

[34] Jose J, Kramer J, Klauser T, Pohlner J, Meyer TF (1996). Absence of periplasmic DsbA oxidoreductase facilitates export of cysteine-containing passenger proteins to the *Escherichia coli* cell surface via the Iga beta autotransporter pathway.. Gene.

[35] Bumann D (2002). Examination of salmonella gene expression in an infected mammalian host using the green fluorescent protein and two-colour flow cytometry.. Mol Microbiol.

[36] Dieckmann-Schuppert A, Schnittler HJ (1997). A simple assay for quantification of protein in tissue sections, cell cultures, and cell homogenates, and of protein immobilized on solid surfaces.. Cell Tissue Res.

[37] Taswell C (1981). Limiting dilution assays for the determination of immunocompetent cell frequencies. I. Data analysis.. J Immunol.

[38] Smelt SC, Cotterell SE, Engwerda CR, Kaye PM (2000). B cell-deficient mice are highly resistant to *Leishmania donovani* infection, but develop neutrophil-mediated tissue pathology.. J Immunol.

[39] Beattie L, Phillips R, Brown N, Owens BM, Chauhan N (2010). IRF-7 contributes to the control of *Leishmania donovani* in the mouse liver.. Infect Immun.

[40] Sabarth N, Hurwitz R, Meyer TF, Bumann D (2002). Multiparameter selection of *Helicobacter pylori* antigens identifies two novel antigens with high protective efficacy.. Infect Immun.

[41] Basu R, Roy S, Walden P (2007). HLA class I-restricted T cell epitopes of the kinetoplastid membrane protein-11 presented by *Leishmania donovani*-infected human macrophages.. J Infect Dis.

[42] Basu R, Bhaumik S, Basu JM, Naskar K, De T (2005). Kinetoplastid membrane protein-11 DNA vaccination induces complete protection against both pentavalent antimonial-sensitive and -resistant strains of *Leishmania donovani* that correlates with inducible nitric oxide synthase activity and IL-4 generation: evidence for mixed Th1- and Th2-like responses in visceral leishmaniasis.. J Immunol.

[43] Rollenhagen C, Sorensen M, Rizos K, Hurvitz R, Bumann D (2004). Antigen selection based on expression levels during infection facilitates vaccine development for an intracellular pathogen.. Proc Natl Acad Sci U S A.

[44] Hess J, Gentschev I, Miko D, Welzel M, Ladel C (1996). Superior efficacy of secreted over somatic antigen display in recombinant Salmonella vaccine induced protection against listeriosis.. Proc Natl Acad Sci U S A.

[45] Kanaly ST, Nashleanas M, Hondowicz B, Scott P (1999). TNF receptor p55 is required for elimination of inflammatory cells following control of intracellular pathogens.. J Immunol.

[46] Sakthianandeswaren A, Elso CM, Simpson K, Curtis JM, Kumar B (2005). The wound repair response controls outcome to cutaneous leishmaniasis.. Proc Natl Acad Sci U S A.

[47] Handman E, Elso C, Foote S (2005). Genes and susceptibility to leishmaniasis.. Adv Parasitol.

[48] Snapper CM, Paul WE (1987). Interferon-gamma and B cell stimulatory factor-1 reciprocally regulate Ig isotype production.. Science.

[49] Trujillo C, Ramirez R, Velez ID, Berberich C (1999). The humoral immune response to the kinetoplastid membrane protein-11 in patients with American leishmaniasis and Chagas disease: prevalence of IgG subclasses and mapping of epitopes.. Immunol Lett.

[50] Rosenzweig D, Smith D, Opperdoes F, Stern S, Olafson RW (2008). Retooling Leishmania metabolism: from sand fly gut to human macrophage.. FASEB J.

[51] Stober CB (2004). From genomes to vaccines for leishmaniasis.. Methods Mol Biol.

[52] Almeida R, Norrish A, Levick M, Vetrie D, Freeman T (2002). From genomes to vaccines: Leishmania as a model.. Philos Trans R Soc Lond B Biol Sci.

[53] Aebischer T, Wolfram M, Patzer SI, Ilg T, Wiese M (2000). Subunit vaccination of mice against new world cutaneous leishmaniasis: comparison of three proteins expressed in amastigotes and six adjuvants.. Infect Immun.

[54] Overath P, Aebischer T (1999). Antigen presentation by macrophages harboring intravesicular pathogens.. Parasitol Today.

[55] Forgber M, Basu R, Roychoudhury K, Theinert S, Roy S (2006). Mapping the antigenicity of the parasites in *Leishmania donovani* infection by proteome serology.. PLoS One.

[56] Forgber M, Gellrich S, Sharav T, Sterry W, Walden P (2009). Proteome-based analysis of serologically defined tumor-associated antigens in cutaneous lymphoma.. PLoS One.

[57] Skeiky YA, Guderian JA, Benson DR, Bacelar O, Carvalho EM (1995). A recombinant Leishmania antigen that stimulates human peripheral blood mononuclear cells to express a Th1-type cytokine profile and to produce interleukin 12.. J Exp Med.

[58] Goto Y, Coler RN, Guderian J, Mohamath R, Reed SG (2006). Cloning, characterization, and serodiagnostic evaluation of *Leishmania infantum* tandem repeat proteins.. Infect Immun.

[59] Peters C, Aebischer T, Stierhof YD, Fuchs M, Overath P (1995). The role of macrophage receptors in adhesion and uptake of *Leishmania mexicana* amastigotes.. J Cell Sci.

[60] Deak E, Jayakumar A, Cho KW, Goldsmith-Pestana K, Dondji B (2010). Murine visceral leishmaniasis: IgM and polyclonal B-cell activation lead to disease exacerbation.. Eur J Immunol.

[61] Bhaumik S, Basu R, Sen S, Naskar K, Roy S (2009). KMP-11 DNA immunization significantly protects against *L. donovani* infection but requires exogenous IL-12 as an adjuvant for comparable protection against *L. major*.. Vaccine.

[62] Nascimento E, Fernandes DF, Vieira EP, Campos-Neto A, Ashman JA (2010). A clinical trial to evaluate the safety and immunogenicity of the LEISH-F1+MPL-SE vaccine when used in combination with meglumine antimoniate for the treatment of cutaneous leishmaniasis.. Vaccine.

[63] Moreno J, Nieto J, Masina S, Canavate C, Cruz I (2007). Immunization with H1, HASPB1 and MML Leishmania proteins in a vaccine trial against experimental canine leishmaniasis.. Vaccine.

[64] Miret J, Nascimento E, Sampaio W, Franca JC, Fujiwara RT (2008). Evaluation of an immunochemotherapeutic protocol constituted of N-methyl meglumine antimoniate (Glucantime) and the recombinant Leish-110f+MPL-SE vaccine to treat canine visceral leishmaniasis.. Vaccine.

[65] Palatnik-de-Sousa CB, Silva-Antunes I, Morgado AA, Menz I, Palatnik M (2009). Decrease of the incidence of human and canine visceral leishmaniasis after dog vaccination with Leishmune in Brazilian endemic areas.. Vaccine.

[66] Galen JE, Pasetti MF, Tennant S, Ruiz-Olvera P, Sztein MB (2009). *Salmonella enterica* serovar Typhi live vector vaccines finally come of age.. Immunol Cell Biol.

[67] Aebischer T, Walduck A, Schroeder J, Wehrens A, Chijioke O (2008). A vaccine against *Helicobacter pylori*: towards understanding the mechanism of protection.. Int J Med Microbiol.

[68] Salerno-Goncalves R, Sztein MB (2009). Priming of *Salmonella enterica* serovar Typhi-specific CD8^+^ T cells by suicide dendritic cell cross-presentation in humans.. PLoS One.

[69] Schroeder J, Aebischer T (2011). Vaccines for Leishmaniasis: From proteome to vaccine candidates.. Hum Vaccin.

[70] Angelakopoulos H, Hohmann EL (2000). Pilot study of phoP/phoQ-deleted *Salmonella enterica* serovar Typhimurium expressing *Helicobacter pylori* urease in adult volunteers.. Infect Immun.

[71] Petavy AF, Hormaeche C, Lahmar S, Ouhelli H, Chabalgoity A (2008). An oral recombinant vaccine in dogs against *Echinococcus granulosus*, the causative agent of human hydatid disease: a pilot study.. PLoS Negl Trop Dis.

[72] McVey DS, Chengappa MM, Mosier DE, Stone GG, Oberst RD (2002). Immunogenicity of chi4127 phoP- *Salmonella enterica* serovar Typhimurium in dogs.. Vaccine.

[73] Chabalgoity JA, Moreno M, Carol H, Dougan G, Hormaeche CE (2000). *Salmonella typhimurium* as a basis for a live oral *Echinococcus granulosus* vaccine.. Vaccine.

