# Supplementary figures and images for: Single Dose Novel Salmonella Vaccine Enhances Resistance against Visceralizing L. major and L. donovani Infection in Susceptible BALB/c Mice
Source: PLoS Negl Trop Dis. 2011 Dec 27;5(12):e1406. doi: 10.1371/journal.pntd.0001406 (PMC3246433; doi:10.1371/journal.pntd.0001406)

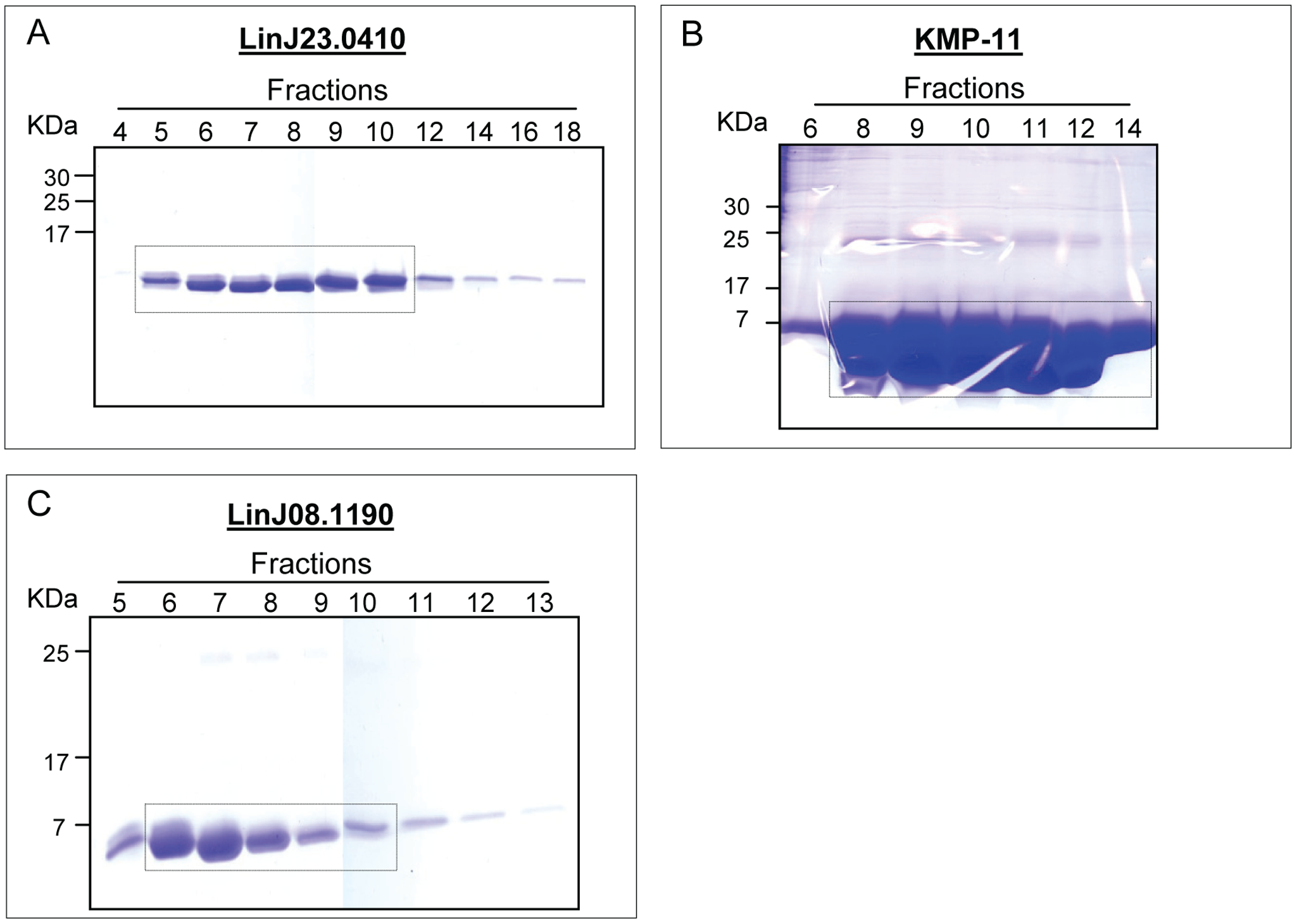

Supplement: Figure S1 — Post-column fractions of purified his-tagged antigens. His-tagged Leishmania antigens were expressed and purified from E. coli cell from inclusion bodies (LinJ23.0410 (A); LinJ08.1190 (B)) or lysates (KMP11 (C)) using nickel column liquid chromatography as described in Materials and Methods. Collected fractions were separated on SDS-Gels to determine yield and purity and selected fractions (box) were pooled and subsequently subjected to downstream processing such as dialysis and ultrafiltration. (TIF) [file pntd.0001406.s001.tif]

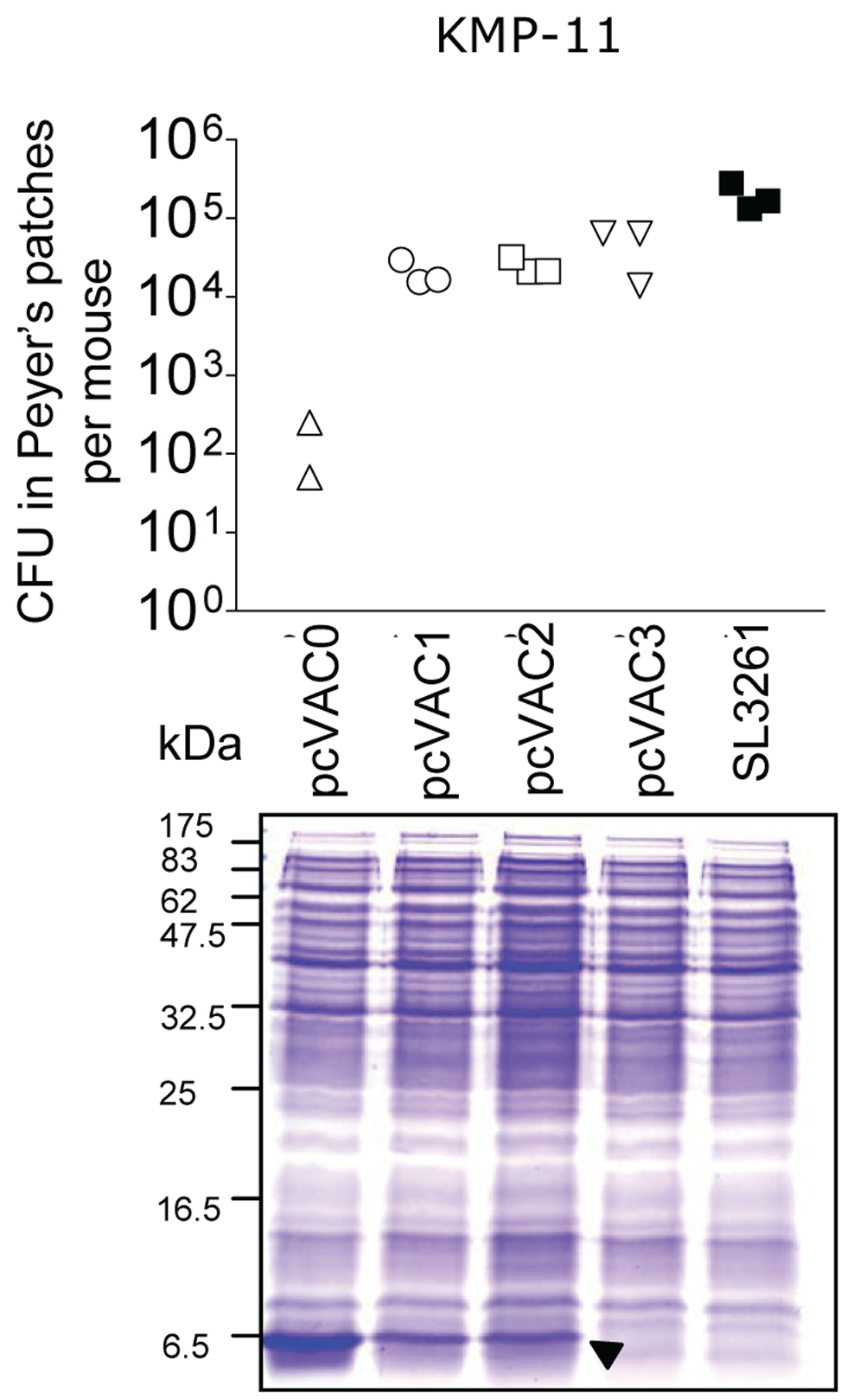

Supplement: Figure S2 — Two-step strategy for the selection of vaccine strains expressing cytosolic KMP-11 for in vivo testing. Bacterial fitness (top panel) was determined as ability of the vaccine strain to colonize the Peyer's patches of mice seven days after single dose oral administration of 1010 CFU with KMP-11 expressing SL3261 (open symbols) or carrier SL3261 control (closed symbol; Schroeder and Aebischer (32)). For antigen expression (bottom panel), bacterial strains were grown under conditions mimicking the intraphagosomal environment (low Mg2+, Ca2+) and thus activating PpagC promoter activated vaccine antigen expression. Translational efficiency depended on ribosomal binding site sequences that were the only difference between expression plasmids pcVAC0-3. 20 µg whole bacterial lysate per strain was loaded onto SDS gels and bands were visualized by coomassie stain. Black arrowhead indicates induced protein of interest (KMP-11). (TIF) [file pntd.0001406.s002.tif]

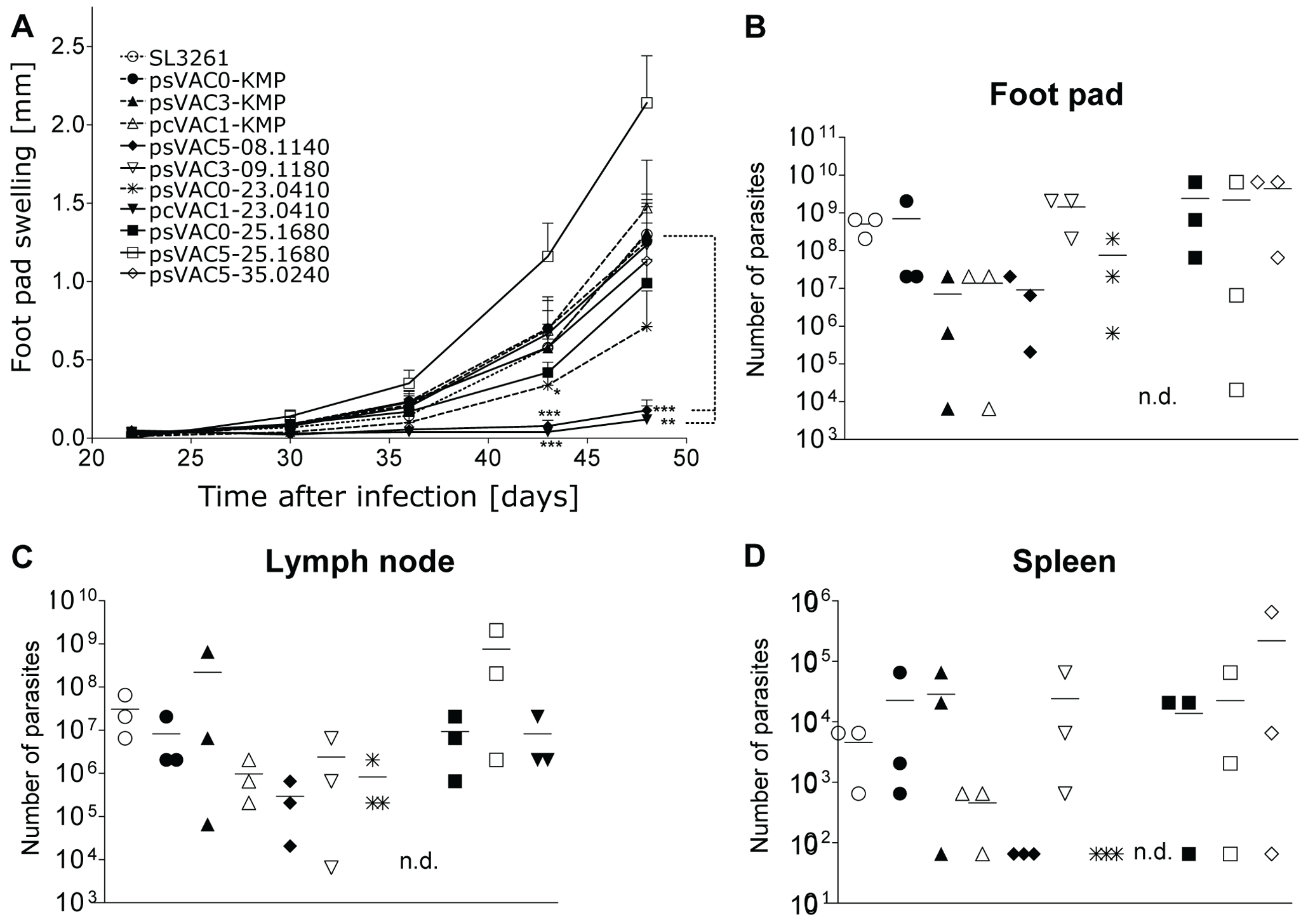

Supplement: Figure S3 — Pilot-study to identify vaccine strains protective against L. major infection. Mice (10 per group) were immunised orally with a single dose of 1010 CFU of Salmonella vaccine strains carrying the indicated vaccine antigen expression plasmids or the carrier control SL3261. Twelve weeks later animals were challenged with 2×10^6^ L. major promastigotes into the left hind foot pad. Lesion size (A) was monitored with a calliper using the uninfected right foot as reference. Values represent mean swelling in mm and bars show standard errors of the mean (SEM). Asterisks denote statistical thresholds * P≤0.05, ** P≤0.01, *** P≤0.001 determined by two-tailed Mann-Whitney U test. Seven weeks after infection, three mice per group were randomly selected for determination of parasite burden in the foot pad (B), draining lymph node (D and spleen (C) were by limiting dilution assay as described in Materials and Methods. n.d. denotes not determined. (TIF) [file pntd.0001406.s003.tif]
